# MicroRNA-22 inhibits tumor growth and metastasis in gastric cancer by directly targeting MMP14 and Snail

**DOI:** 10.1038/cddis.2015.297

**Published:** 2015-11-26

**Authors:** Q-F Zuo, L-Y Cao, T Yu, L Gong, L-N Wang, Y-L Zhao, B Xiao, Q-M Zou

**Affiliations:** 1 National Engineering Research Center of Immunological Products, Department of Microbiology and Biochemical Pharmacy, College of Pharmacy, Third Military Medical University, Chongqing 400038, China; 2 General Surgery and Center of Minimally Invasive Gastrointestinal Surgery, Department of General Surgery, Southwest Hospital, Third Military Medical University, Chongqing 400038, China

## Abstract

MicroRNAs (miRNAs) deregulation is frequent in human gastric cancers (GCs), but the role of specific miRNAs involved in this disease remains elusive. MiR-22 was previously reported to act as tumor suppressors or oncogenes in diverse cancers. However, their accurate expression, function and mechanism in GC are largely unclear. Here, we found that the expression of miR-22 was significantly reduced in clinical GC tissues compared with paired adjacent normal tissues, and was significantly correlated with a more aggressive phenotype of GC in patients, and miR-22 low expression correlated with poor overall survival. The introduction of miR-22 markedly suppressed GC cell growth, migration and invasion, and inhibition of miR-22 promoted GC cell proliferation, migration and invasion *in vitro*. We further demonstrated that miR-22 acted as tumor suppressors through targeting extracellular matrix (ECM) remodeling member matrix metalloproteinase 14 (MMP14) and epithelial-to-mesenchymal transition (EMT) inducer Snail in GC. Moreover, ectopic expression of MMP14 or Snail restored inhibitory effects of miR-22 on cell migration and invasion in GC cells, and a negative relationship between the miR-22 expression and MMP14 or Snail mRNA levels was observed in GC. Finally, overexpression of miR-22 suppressed tumor growth, peritoneal dissemination and pulmonary metastasis *in vivo*. Taken together, we identified that miR-22 is a potent tumor suppressor in GC. MiR-22 downregulation promotes GC invasion and metastasis by upregulating MMP14 and Snail, and then inducing ECM remodeling and EMT. These findings provide a better understanding of the development and progression of GC and may be an important implication for future therapy of the GC.

Gastric cancer (GC) is the fifth most prevalent type of malignancy and the third leading cause of cancer death worldwide. It is estimated that 951 600 new stomach cancer cases and 723 100 deaths occurred in 2012.^1^ In most countries, survival from stomach cancer remained in the narrow range of 25–30%.^2^ Although therapeutic methods are improving in surgical combined with radiotherapy and chemotherapy, the prognosis for advanced stage patients is still very poor.^3, 4^ Thus, an improved and detailed understanding of the precise molecular mechanisms underlying GC development and progression is urgently needed.

MicroRNAs (miRNAs) are small noncoding RNAs, which lead to silencing of their cognate target genes by either inhibiting their translation or degrading mRNA molecules.^5^ These large families of highly conserved miRNAs have been implicated in the regulation of a variety of GC progression, including growth and metastasis, implying that they can function either as tumor suppressors or oncogenes.^6, 7, 8^ For instance, miR-375 frequently downregulated in GC inhibits cell proliferation by targeting Janus kinase 2.^9^ MiR-124 and miR-206 inhibit cell proliferation in GC through downregulation of sphingosine kinase 1 and by repressing cyclin D2, respectively.^10^ Recent studies demonstrate that various miRNAs are involved in GC metastatic processes.^11, 12^ MiR-495 and miR-551a inhibit the migration and invasion of human GC cells by directly interacting with phosphatase of regenerating liver-3.^13^ MiR-7 is significantly downregulated in highly metastatic GC cell lines and metastatic tissues, and miR-7 downregulated Snail, through targeting insulin-like growth factor 1 receptor, and suppressed E-cadherin expression and inhibited epithelial-to-mesenchymal transition (EMT) in GC cells.^14^ Our previous studies showed that miR-141 inhibited GC growth and metastasis by directly targeting transcriptional co-activator with PDZ-binding motif, and miR-25 promoted GC migration, invasion and proliferation by directly targeting transducer of ERBB2 1 (TOB1) and correlated with poor survival.^15, 16^ Above studies suggested a close correlation between miRNAs and the development, progression, metastasis and prognosis of GC. Previous studies have shown that miR-22 functioned in multiple cellular processes and their deregulation is a hallmark of human cancer.^17^ Despite an increasing number of studies on the biogenesis and mechanisms of miR-22 in the pathogenesis of GC,^18, 19^ the accurate expression and mechanistic function of them in GC remain elusive.

Metastasis is the most significant process affecting the clinical management of cancer patients and occurs in multiple sequential steps. One intrinsic property of metastatic tumor cells that allows them to breach tissue barriers is their ability to degrade the proteins of the extracellular matrix (ECM). This ECM remodeling by cancer cells depends on matrix metalloproteinases (MMPs).^20, 21^ MMP14 is one of the membrane-anchored MMPs and it has a central role in tumor invasion and not only degrades the ECM but also promotes the secretion of pro-MMP2 and pro-MMP9.^22^ Previous studies showed that MMP14 is elevated in GC patients, and MMP14 overexpression is closely associated with GC invasion.^23, 24^ EMT describes the molecular reprogramming and phenotypic changes characterizing the conversion of polarized immotile epithelial cells to motile mesenchymal cells.^25, 26^ This process promotes tumor invasion and metastasis. One of the hallmarks of EMT is the functional loss of E-cadherin, which is thought to be a metastatic suppressor during tumor progression.^27^ As a key transcriptional repressor of E-cadherin expression in EMT, Snail has an important role in cancer progression.^28^ Previous studies reported that overexpression of Snail is associated with lymph node metastasis and Snail is an independent prognostic predictor for progression and patient survival of GC.^29, 30^

Here, we identified miR-22 as one of the most significantly downregulated miRNAs in GC tissues and a critical suppressor of GC cell proliferation, invasion and metastasis both *in vitro* and *in vivo* studies. Importantly, we demonstrated that miR-22 downregulation promoted invasion and metastasis through inducing ECM remodeling and EMT by directly targeting MMP14 and Snail.

## Results

### MiR-22 is downregulated in primary tumor tissues of GC and downregulation of miR-22 is correlated with GC progression and poor survival

**Figure 1:**
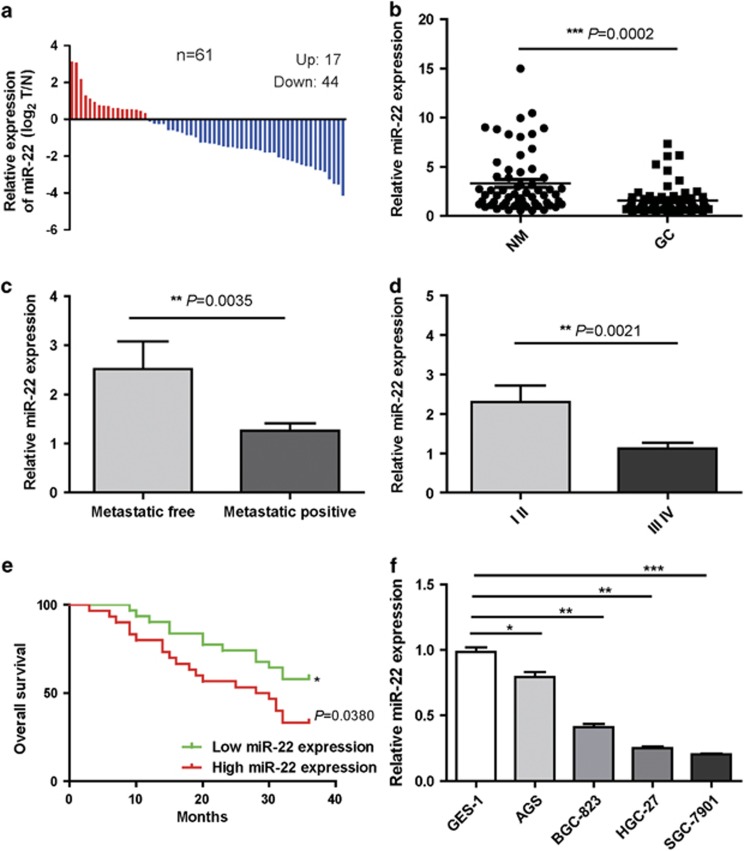
MiR-22 is downregulated in primary tumor tissues of GC and correlates inversely with metastatic capacity in GC tissue. (**a**) Relative levels of miR-22 in 61 surgical specimens of GC and matched adjacent nonmalignant tissues were quantified by qRT-PCR. Data were presented as log2 fold change (ΔΔCt values, GC/nonmalignant, T/N). (**b**) Expression status of miR-22 in an independent validation cohort of 61 pairs of matching GC and NM tissues (paired *t*-test; ***P<0.001). (**c**) Means of miR-22 relative levels from GC tissues including a group of 46 GC patients with positive lymph node metastases compared with another group of 15 GC patients with negative lymph node metastases (**P<0.01). (**d**) Correlation of miR-22 expression with clinicopathological stage of GC tissues used for miR-22 expression analysis (**P<0.01). (**e**) Kaplan–Meier survival analysis of overall survival duration for two groups defined by low and high expression of miR-22 in patients with GC. The log-rank test was used to calculate *P*-values (*P<0.05). (**f**) Expression levels of miR-22 in normal gastric mucous epithelium cell (GES-1) and four GC cells (AGS, BGC-823, HGC-27 and SGC-7901). Data are presented as mean±S.D. RNU6B serve as an internal reference. All assays were performed in duplicate. *P<0.05, **P<0.01, ***P<0.001

To identify the roles of miR-22 in the development of GC, we analyzed the expression level of miR-22 in 61 pairs of frozen GCs and matched adjacent normal mucosa (NM) tissues by quantitative real-time PCR (qRT-PCR). The qRT-PCR analyses showed that the expression of miR-22 was reduced in 44 of 61 (72%) tumor samples compared with their nonmalignant counterparts (Figure 1a). The average expression level of miR-22 was significantly decreased in tumor tissues compared with paired NM tissues (*P*<0.001; Figure 1b). To further investigate the clinicopathological and prognostic significance of miR-22 levels in patients with GC, the levels of miR-22 in 61 pairs of GC tissues were statistically analyzed. When the 61 tumors were stratified, based on clinicopathological features, we found that miR-22 expression was significantly decreased in primary tumors that subsequently metastasized compared with those that did not metastasize (*P*<0.01; Figure 1c), and low-level expression of miR-22 in GC was significantly associated with a more aggressive tumor phenotype (*P*< 0.01, stage I/II *versus* III/IV; Figure 1d). Kaplan–Meier analysis on patients with survival data revealed that miR-22 low expression correlated with poor overall survival (*P*<0.05; Figure 1e). We also determined the expression of miR-22 in normal gastric mucous epithelium cell (GES-1) and four GC cells (AGS, BGC-823, HGC-27 and SGC-7901). It was shown that miR-22 was downregulated in GC cells (AGS, BGC-823, HGC-27 and SGC-7901) compared with normal gastric mucous epithelium cell (GES-1) (Figure 1f). Our results indicate that miR-22 is downregulated in GCs and downregulation of miR-22 is correlated with GC progression and poor survival, and suggest an invasion and metastasis inhibition function of miR-22 in GC.

### Ectopic expression of miR-22 suppresses cell proliferation, migration and invasion in GC cells *in vitro*

**Figure 2:**
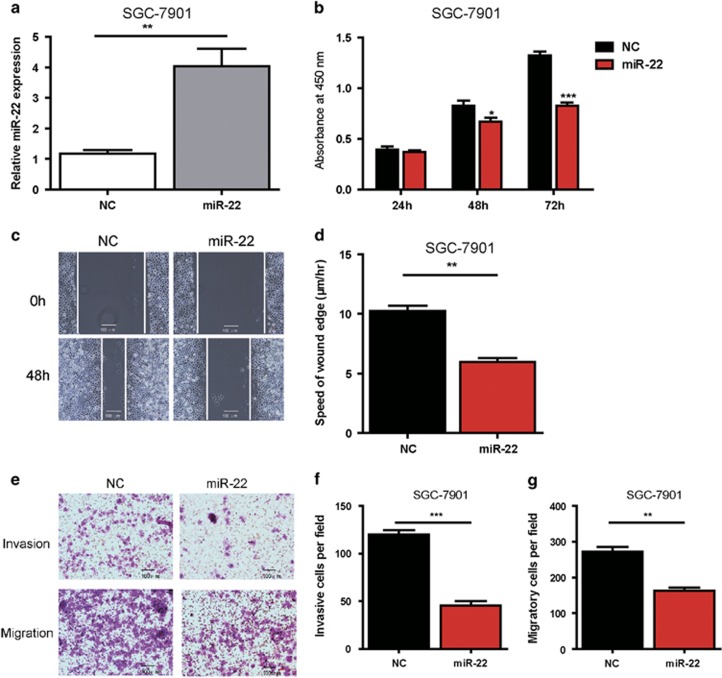
Ectopic expression of miR-22 suppresses cell proliferation, migration and invasion in SGC-7901 cells *in vitro*. (**a**) After SGC-7901 cells were transfected with miR-NC (50 nM), miR-22 (50 nM) for 24 h, the efficacy of miR-22 overexpression in SGC-7901 was determined by qRT-PCR. Data are presented as mean±S.D. (*n*=3). (**b**) Effect of miR-22 (50 nM) and controlled miR-NC (50 nM) on SGC-7901 cells proliferation was measured by CCK8 assay at 24, 48, 72-h posttransfection. Absorbance was read at 450 nm. Data are presented as mean±S.D. (*n*=6; *P<0.05, **P<0.01). (**c** and **d**) Representative photomicrographs of wound-healing assays results for SGC-7901 cells transfected with miR-22 (50 nM) or miR-NC (50 nM) at 0 and 48- h posttransfection. Wound healing was quantified by measurement of the average linear speed of movement of the wound edges. All of the experiments were performed three times. Data are presented as mean±S.D. (*n*=5) (× 200 magnification, **P<0.01). Representative images (**e**) and bar graphs (**f** and **g**) depicting the invasion and migration ability of SGC-7901 after miR-NC (50 nM), or miR-22 (50 nM) transfection. Data are presented as mean±S.D. (*n*=5) and analyzed by a two-tailed unpaired *t*-test (× 200 magnification, **P<0.01, ***P<0.001)

To better understand the mechanistic role of miR-22 in gastric carcinogenesis, we performed miR-22 overexpression experiments by miR-22 transfection in the SGC-7901 cell line, in which miR-22 expression was lowest among four GC cell lines (Figure 1f). Overexpression of miR-22 was confirmed by qRT-PCR, as shown in Figure 2a. To determine the role of miR-22 in the proliferation of GC cells *in vitro*, CCK8 assay were performed, as shown in Figure 2b. Overexpression of miR-22 inhibited cell proliferation in SGC-7901 at 48 h, 72 h after transfection. Then, we analyzed the effect of ectopic miR-22 expression on cellular invasion and migration potential of SGC-7901 cells. In the wound-healing assay, high miR-22 expression significantly suppressed the ability of cells to migrate (Figures 2c and d). In the transwell invasion and migration assay, cells transfected with miR-22 mimics displayed an inhibition in invasion and migration ability when compared with the control group in SGC-7901 cells (Figures 2e–g). Collectively, these results indicated that ectopic miR-22 significantly suppressed cell proliferation, migration and invasion *in vitro*.

### Inhibition of miR-22 promotes cell proliferation, invasion and migration in GC cells *in vitro*

**Figure 3:**
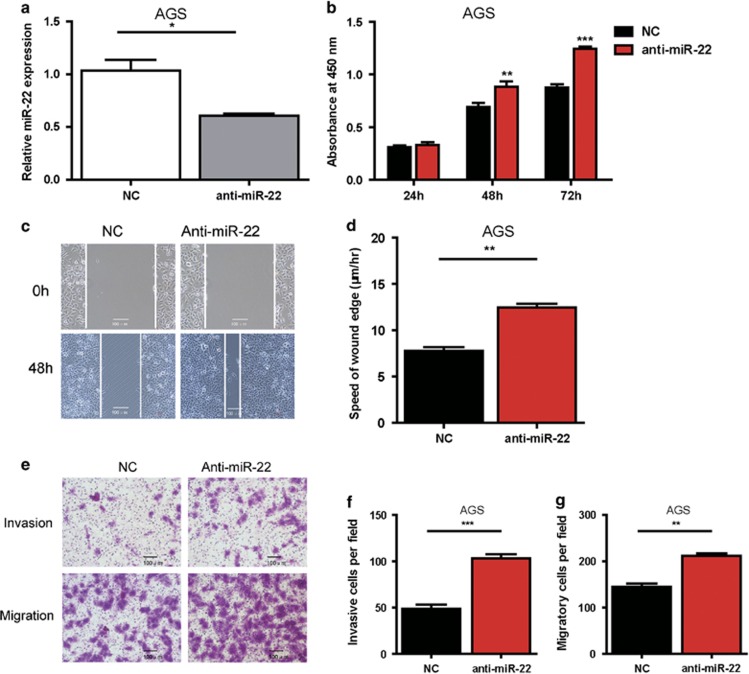
Inhibition of miR-22 promotes cell proliferation, migration and invasion in AGS cells *in vitro*. (**a**) After AGS cells were transfected with miR-NC (50 nM), anti-miR-22 (50 nM) for 24 h, the efficacy of miR-22 inhibition in AGS was determined by qRT-PCR. Data are presented as mean±S.D. (*n*=3; *P<0.05). (**b**) Effect of anti-miR-22 (50 nM) and controlled miR-NC (50 nM) on AGS cells proliferation was measured by CCK8 assay at 24, 48, 72- h posttransfection. Absorbance was read at 450 nm. Data are presented as mean±S.D. (*n*=6). (**c** and **d**) Representative photomicrographs of wound-healing assays results for AGS cells transfected with anti-miR-22 (50 nM) or miR-NC (50 nM) at 0 and 48- h posttransfection. Wound healing was quantified by measurement of the average linear speed of movement of the wound edges. All of the experiments were performed three times. Data are presented as mean±S.D. (*n*=5) (× 200 magnification, **P<0.01). Representative images (**e**) and bar graphs (**f** and **g**) depicting the invasion and migration ability of AGS after miR-NC (50 nM), or anti-miR-22 (50 nM) transfection. Data are presented as mean±S.D. (*n*=5) and analyzed by a two-tailed unpaired *t*-test (× 200 magnification, **P<0.01, ***P<0.001)

To be logical and direct demonstration of the consequences of the low expression levels of miR-22 in GC, we performed miR-22 inhibition experiments by anti-miR-22 transfection in the AGS cell line, in which miR-22 expression was higher than other three GC cell lines (Figure 1f). MiR-22 was transiently inhibited in AGS cells with anti-miR-22 (Figure 3a). Then, we analyzed the effect of inhibition of miR-22 levels on cellular proliferation, invasion and migration potential of AGS cells. The results showed that the suppression of miR-22 enhanced cell proliferation (Figure 3b), invasion and migration (Figures 3c–g).

### MiR-22 directly regulates MMP14 and Snail in GC cells

**Figure 4:**
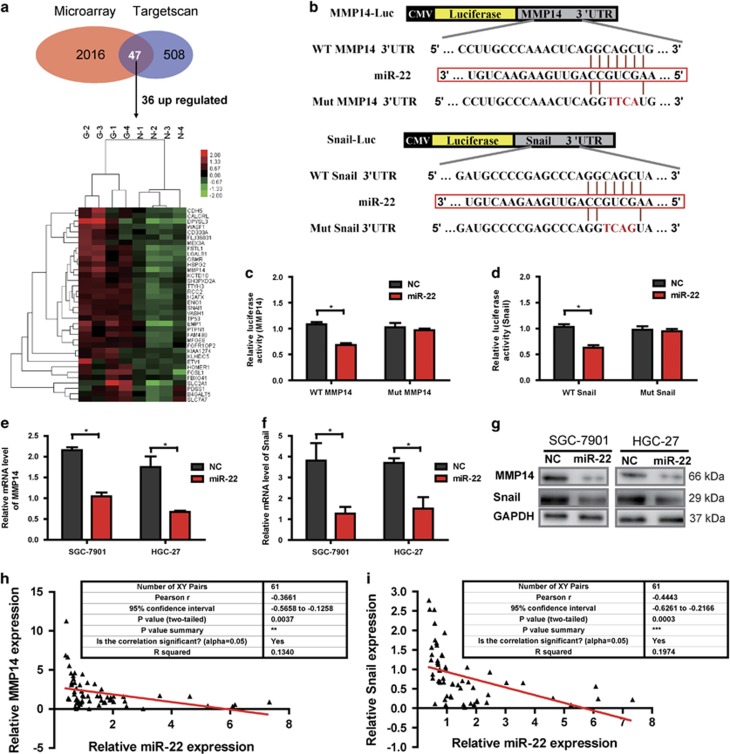
Prediction and validation of miR-22 target gene in GC cells. (**a**) Thirty-six target genes, which have miR-22 seed sites, were predicted via miRNA target prediction tools (TargetScan) and microarray analysis (upregulated expression of protein-coding genes between paired GC and normal mucosa tissues). (**b**) The sequence of miR-22 (middle) matches the 3′UTR of MMP14 and 3′UTR of Snail (top). Bottom, mutations of the 3′UTR of MMP14 and 3′UTR of Snail. (**c** and **d**) MiR-22 inhibited wild-type, but not mutated MMP14 3′UTR and Snail 3′UTR luciferase reporter activity. HEK293T cells were co-transfected with firefly luciferase reporter plasmids containing wild-type or mutant MMP14 3′UTR and Snail 3′UTR, and pRL-TK plasmid and miR-22 mimics or miR-NC as indicated. After 24 h, firefly luciferase activities were measured and normalized by use of renilla luciferase activities. Data were presented as as mean±S.D. (*n*=6). *P<0.05. (**e** and **f**) The relative MMP14 and Snail mRNA levels determined by qRT-PCR in miR-22 or NC transfected SGC-7901 and HGC-27 cells (*P<0.05). (**g**) Overexpression of miR-22 suppressed protein expression (western blotting) of MMP14 and Snail in SGC-7901 and HGC-27 cells. GAPDH was used as internal control and was also detected by western blotting. **(h)** Scatter shows that the inverse correlation between the expression levels of miR-22 and MMP14 mRNA in the 61 gastric tumor tissues (*r*=–0.3661; **P<0.01). **(i)** Scatter shows that the inverse correlation between the expression levels of miR-22 and Snail mRNA in the 61 gastric tumor tissues (*r*=–0.4443; ***P<0.001)

As the function of miRNAs in tumor development is dependent on targeting their key target genes, it is crucial important to identify the targets of miR-22. Candidate targets were first determined using target prediction engine TargetScan. We next examined the mRNAs expression profile in four pairs of primary tumor tissues of GC patients with and matched non-tumor tissues by microarray analysis. The results showed that 2016 mRNAs were differently regulated (fold change ⩾1.5 and a *P*-value ⩽0.05; data not shown). When compared with the predicted targets by TargetScan, 36 target genes, which have miR-22 seed sites, were screened via TargetScan and microarray analysis (Figure 4a). Among these candidate targets, MMP14 and Snail were predicted as novel targets of miR-22 and were selected as our target genes in GC for further study, as they have been shown to associate with prognosis and metastasis in patients with GCs.^23, 24, 29, 30^ To determine whether MMP14 and Snail are direct targets of miR-22, wild-type and mutant 3′ untranslated regions (3′UTRs) of MMP14 and Snail were cloned into the downstream of firefly luciferase coding region in pGL-3 luciferase reporter vector. The constructs were then co-transfected with pRL-TK and miR-22 mimics or miR-NC into HEK293T cells, respectively. The relative luciferase activity was reduced by 37% and 39% in pGL-3 vectors with wild-type MMP14 and Snail 3′UTRs, respectively, but not in those with respective mutant 3′UTRs (Figures 4b–d). To further determine whether miR-22 can decrease endogenous MMP14 and Snail expression, we transfected miR-22 mimics in SGC-7901 and HGC-27 cells. As shown in Figures 4e–g, overexpression of miR-22 resulted in significant reduction in MMP14 and Snail mRNA transcription as well as protein expression. These results suggested that MMP14 and Snail could be direct targets of miR-22. As MMP14 and Snail transcripts were identified as direct targets of miR-22, we examined the relationship between their mRNA expression and miR-22 expression in the 61 GC tissues using qRT-PCR. The results showed that the mRNA levels of MMP14 or Snail were inversely correlated with miR-22 levels in the 61 primary GC tissues (Figures 4h and i). In summary, negative regulation of MMP14 and Snail by miR-22 is clinically relevant in the context of GC.

### *In vitro* functional analysis and expression of MMP14 and Snail in GC cells, and ectopic expression of MMP14 or Snail restores inhibitory effects of miR-22 on cell migration and invasion in GC cells

**Figure 5:**
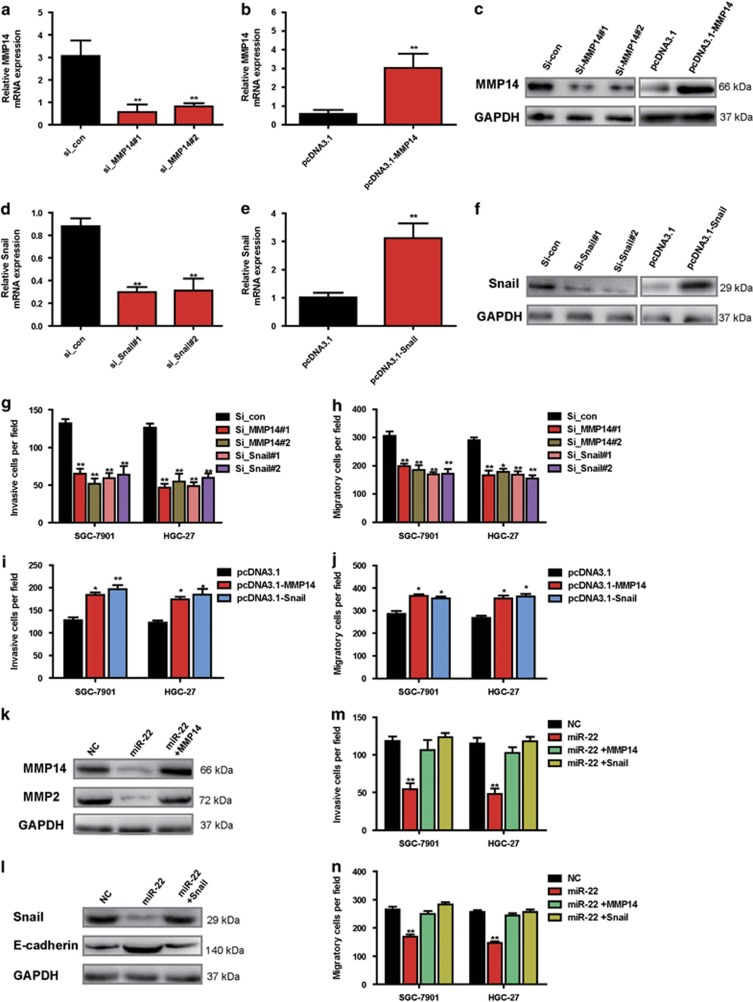
*In vitro* functional analysis and expression of MMP14 and Snail in GC cells, and ectopic expression of MMP14 or Snail restores the effects of miR-22 on cell migration and invasion in GC cells. (**a**, **b**, **d** and **e**) qRT-PCR assays show the mRNA expression of MMP14 and Snail in GC cells transfected with si_con, si_MMP14#1, si_MMP14#2, si_Snail#1, si_Snail#2, pcDNA3.1 vector, pcDNA3.1-MMP14 vector, or pcDNA3.1-Snail vector, respectively. Data are presented as mean±S.D. (*n*=3). **P<0.01. (**c** and **f**) Western blotting assays show the protein expression of MMP14 and Snail in GC cells transfected with si_con, si_MMP14#1, si_MMP14#2, si_Snail#1, si_Snail#2, pcDNA3.1 vector, pcDNA3.1-MMP14 vector, or pcDNA3.1-Snail vector. GAPDH served as an internal reference. (**g** and **h**) Inhibition of cell migration and invasion by knockdown of MMP14 and Snail. SGC-7901 and HGC-27 cells were transfected with si_con, si_MMP14#1, si_MMP14#2, si_Snail#1, or si_Snail#2, respectively. The assays were repeated three times. Data are presented as mean±S.D. (*n*=5). **P<0.01. (**i** and **j**) Promotion of cell migration and invasion by overexpression of MMP14 or Snail. SGC-7901 and HGC-27 cells were transfected with pcDNA3.1 vector, pcDNA3.1-MMP14 vector, or pcDNA3.1-Snail vector, respectively. The assays were repeated three times. Data are presented as mean±S.D. (*n*=5). *P<0.05, **P<0.01. (**k**) Western blot assays show the protein expression of MMP14 and MMP2 in GC cells after transfection with miR-NC, miR-22 mimics, or co-transfection with miR-22 mimics and pcDNA3.1-MMP14 vector. GAPDH served as an internal reference. (**l**) Western blot assays show the protein expression of Snail and E-cadherin in GC cells after transfection with miR-NC, miR-22 mimics, or co-transfection with miR-22 mimics and pcDNA3.1-Snail vector. GAPDH served as an internal reference. (**m** and **n**) MMP14 or Snail overexpression partially rescues miR-22-reduced cell migration and invasion. Data are presented as mean±S.D. (*n*=5). **P<0.05. The assays were repeated in duplicate

MMP14 has been suggested to involve in cancer invasion and metastasis by degrading the ECM and increasing the secretion of pro-MMP2 and pro-MMP9.^31^ Snail has an important role in cancer progression. Emerging evidences indicate that Snail confers tumor cells with cancer stem cell-like traits, and promotes tumor recurrence and metastasis.^28^ To confirm whether downregulation of MMP14 and Snail by miR-22 could result in inhibition of migration and invasion of GC cells, we knocked down the expression of endogenous MMP14 or Snail by their small interfering RNAs (siRNAs) to mimic the effects of miR-22 overexpression. When the mRNA and protein levels of both MMP14 and Snail were significantly reduced by siRNAs in SGC-7901 cell (Figures 5a, c, d and f), invasion and migration of the cells were correspondingly significantly inhibited (Figures 5g and h), suggesting that the inhibitory effects of miR-22 on cells migration and invasion could, at least partially, act through its inhibition of MMP14 and Snail activities. Meanwhile, we evaluated the effects of overexpression of MMP14 or Snail protein with pcDNA3.1-MMP14 or pcDNA3.1-Snail, respectively. The ectopic expression results showed that overexpression of MMP14 or Snail enhanced MMP14 or Snail mRNA and protein levels (Figures 5b, c, e and f), and promoted cell invasion and migration (Figures 5i and j). Moreover, we used SGC-7901 and HGC-27 cells co-transfected with miR-22 and MMP14 or Snail to test whether overexpression of MMP14 or Snail could reverse the inhibitory effects of miR-22 on migration and invasion of GC cells. As predicted, MMP14 and its target MMP2 expression were markedly decreased in the GC cells after transfection with miR-22, and were restored when the GC cells were co-transfected with pcDNA3.1-MMP14 and miR-22 mimics (Figure 5k). Snail expression was markedly decreased and Snail targets E-cadherin was markedly increased in the GC cells after transfection with miR-22, and were restored when the GC cells were co-transfected with pcDNA3.1-MMP14 and miR-22 mimics (Figure 5l). Function investigation showed that the co-transfection of pcDNA3.1-MMP14 or pcDNA3.1-Snail and miR-22 mimics into SGC-7901 and HGC-27 cells significantly reversed miR-22-suppressed migration and invasion (Figures 5m and n). These findings demonstrated that miR-22 inhibited migration and invasion of GC cells via the miR-22/MMP14/Snail signaling axis.

### MiR-22 inhibited the growth of SGC-7901-engrafted tumors and repressed the peritoneal dissemination and distal pulmonary metastases *in vivo*

**Figure 6:**
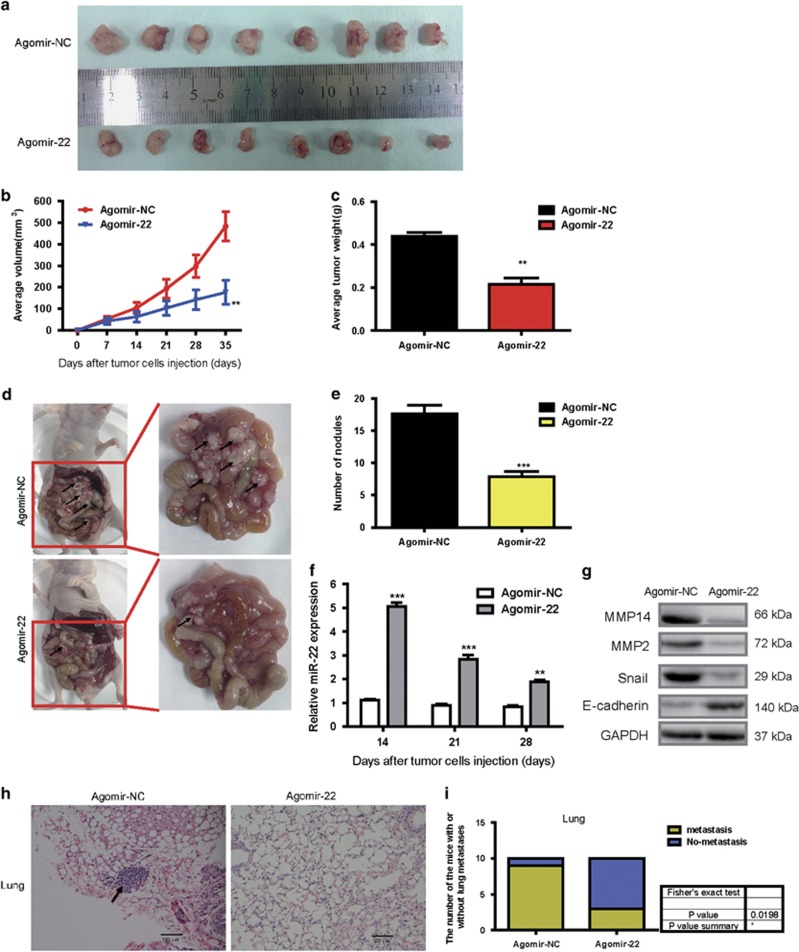
Overexpression of miR-22 inhibits GC growth and represses the peritoneal dissemination and distal pulmonary metastases *in vivo*. (**a**) Photographs of mice injected with agomir-NC-SGC-7901 or agomir-22-SGC-7901. In all, 2 × 10^6^ SGC-7901 cells transfected with agomir-NC (5 *μ*M) or agomir-22 (5 *μ*M), respectively, were suspended in 200 *μ*l phosphate-buffered saline for each mouse and were injected subcutaneously into the axillary fossae of the female nude mice (*n*=8). (**b**) Graph representing tumor volumes at the indicated days during the experiment for the two groups: agomir-NC and agomir-22. Data are presented as mean±S.D. **P<0.01. (**c**) Tumor weight averages between agomir-NC-SGC-7901 and agomir-22-SGC-7901 mice groups at the end of the experiment (day 35). Data are presented as mean±S.D. (*n*=8). **P<0.01. (**d** and **e**) Representative tumor nodules in the abdominal cavity of nude mice that were injected with SGC-7901 cells containing agomir-NC or agomir-22. In all, 1 × 10^6^ SGC-7901 cells transfected with agomir-NC or agomir-22, respectively, were injected into the abdominal cavity of nude mice (*n*=6). Four weeks after injection, the mice were killed and the macroscopic nodules in abdominal cavity of the mice were counted. The arrowheads point to the tumor nodules in the abdominal cavity. Histogram reveals the number of macroscopic nodules. Data are presented as mean±S.D., ***P<0.001. (**f**) qRT-PCR assays determine miR-22 expression in the metastasized nodules (28 days after injection). (**g**) Western blotting assays reveal the protein expression of MMP14, MMP2, Snail and E-cadherin in the metastasized nodules (day 28). GAPDH serves as an internal control. (**h**) HE staining of lung tissue isolated from nude mice (*n*=10), which were injected with 1 × 10^6^ SGC-7901 cells containing agomir-NC (5 *μ*M) or agomir-22 (5 *μ*M) via lateral tail veins (× 200 magnification) (35 days after injection). The metastasis nodules are indicated by arrows. (**i**) The graph gives the incidences of metastasis in mice that had received lateral tail injections of each cell line. Ten mice in each group, Fisher's exact test: *P<0.05

To further investigate the contribution of miR-22 *in vivo*, we selected SGC-7901 cell, which possesses the lowest expression of miR-22 to perform the tumor xenograft studies, peritoneal dissemination and pulmonary metastasis via BALB/c nude mice models. Tumor xenograft studies showed that the volumes of the tumors resulting from agomir-22-SGC-7901 injection were significantly smaller than those resulting from agomir-NC-SGC-7901 (Supplementary Figure S1, Figures 6a and b). In agreement with the tumor volumes, the weights of tumors from agomir-22-SGC-7901 group were significantly lower than agomir-NC-SGC-7901 (Figure 6c). Peritoneal dissemination assays showed that mice injected with agomir-22-transfected SGC-7901 cells exhibited significantly reduced number of macroscopic nodules in the peritoneal cavity (Figures 6d and e) In addition, the qRT-PCR analyses showed miR-22 expression levels in agomir-22-SGC-7901 group were significantly increased compared with agomir-NC-SGC-7901 group, and immunoblot analyses revealed that nodules from agomir-22- SGC-7901 cells had reduced MMP14, MMP2 and Snail protein levels and increased E-cadherin protein levels compared with agomir-22- SGC-7901 cells (Figures 6f and g). MMP2 are known as MMP14 target, which facilitates ECM remodeling.^32^ E-cadherin is known as Snail target, which inhibits EMT and migration and invasion.^33^ For pulmonary metastasis assays, examination of the lungs clearly revealed that the number of mice with lung metastases was lower in the group injected with agomir-22- SGC-7901 cells compared with the group injected with agomir-NC-SGC-7901 cells (Figures 6h and i). Together, the data suggest that miR-22 inhibit the growth of SGC-7901-engrafted tumors and repress the peritoneal dissemination and distal pulmonary metastases *in vivo*.

## Discussion

In this study, qRT-PCR validation results showed that miR-22 expression was significantly decreased in GC tissues compared with the paired adjacent normal tissues. In addition, low-level expression of miR-22 in GC was significantly associated with a more aggressive GC phenotype, and miR-22 low expression correlated with poor overall survival. Furthermore, our results indicated that miR-22 directly targeted ECM remodeling member MMP14 and EMT inducer Snail, leading to repressed cell proliferation and inhibited cell invasion and migration in GC cells. Moreover, our investigation for the expression of MMP14, Snail and miR-22 in 61 GC patients indicated that the mRNA levels of MMP14 or Snail were inversely correlated with miR-22 levels. Importantly, overexpressing miR-22 ameliorated progression of GC in an established experimental xenograft model and repressed the peritoneal dissemination and distal pulmonary metastases *in vivo*. Using a series of *in vitro* and *in vivo* assays, we uncovered that miR-22 act as an important tumor suppressor in the normal gastric mucosa.

Previous studies have suggested that miR-22 functioned in multiple cellular processes, including proliferation, differentiation, senescence and apoptosis, and their deregulation is a hallmark of human cancer.^17^ MiR-22 was identified to be downregulated in diverse cancers, including colon cancer,^34^ hepatocellular carcinoma,^34^ ovarian cancer,^35^ lung cancer,^36^ prostate cancer and esophageal squamous cell carcinoma (ESCC).^37, 38^ Yang *et al.*^38^ showed that miR-22 was significantly downregulated in ESCC tissues and inhibited the ESCC cells migration and invasion *in vitro*. Ling *et al.*^36^ demonstrated that miR-22 suppresses lung cancer cell progression through post-transcriptional regulation of epidermal growth factor receptor 3. In prostate cancer, Pasqualini *et al.*^37^ showed that miR-22 and miR-29a were less abundant in the cancerous tissue compared with the benign counterpart and functioned as tumor suppressors by modulating cancer associated targets LAMC1 and Mcl-1. In colon and liver cancer, Yang *et al.*^34^ demonstrated that miR-22 had a tumor-suppressive effect by inhibiting cyclin A2 expression. Apart from miR-22 functioned as a tumor suppressor, miR-22 acted as a potent proto-oncogenic miRNA precisely because of its ability to epigenetically derange the biology of the cell. Song *et al.*^39^ demonstrated that miR-22 exerted its metastatic potential by silencing antimetastatic miR-200 through direct targeting of the Ten eleven translocation (TET) family of methylcytosine dioxygenases, thereby chromatin remodeling toward miR-200 transcriptional silencing. In a back to back study, Song *et al.* identified miR-22 as a key regulator of the self-renewal machinery of the hematopoietic system. The results showed that miR-22 appeared to be elevated in human MDS and leukemia and its deregulation expression correlated with poor survival of patients and TET2 downregulation.^40^ MiR-22 exhibits complex dysregulation in different circumstances and different subcellular distributions, therefore, miR-22 expression may be oppositely changed in the progressions of different tumors. In GCs, integrative network analysis by Tseng *et al.*^41^ showed that compared with the normal gastric tissues, miR-22 was one of the 23 downregulated miRNAs in cancerous tissues. Guo *et al.*^19^ showed miR-22 was downregulated in GC, and it inhibited cell migration and invasion via targeting transcription factor Sp1. Wang *et al.*^18^ demonstrated that miR-22 suppressed the proliferation and invasion of GC cells by inhibiting CD151. These findings suggest that miR-22 regulate cell proliferation, migration and invasion through different target genes and are thereby intimately involved in the development and progression of GC. Consistent with above findings, our study provides a novel and comprehensive insight into the functional role of miR-22 as it relates to GC development and progression and metastatic processes.

Most importantly, our results established MMP14 and Snail as direct functional effectors of miR-22 in GC. MMP14 is a ‘master switch' proteinase with a C-terminal sequence that acts as membrane-anchoring domain, and is a key enzyme involved in ECM degradation and invasion of tumor cells. MMP14 cleaves a variety of substrates including collagens, cell surface proteins such as CD44 and other MMPs such as pro-MMP2 and pro-MMP13.^42^ All these activated target molecules are implicated in ECM remodeling. MMP14 is upregulated in human cancers, including GC. Dong *et al.*^23^ reported that increased expression of MMP14 correlated with the poor prognosis of Chinese patients with GC. Peña *et al.*^24^ also reported that expression of the MMP14 was a potential molecular marker in advanced human GC. Snail is a prominent inducer of EMT and strongly represses E-cadherin expression. Many studies found expression of Snail positively correlates with tumor grade, metastasis and poor prognosis in various tumors. In GC, Shin *et al.*^29^ and He *et al.*^30^ demonstrated Snail is an independent prognostic predictor for progression and patient survival of GC. Up to now, knowledge of functional roles and regulatory mechanism of MMP14 and Snail in GC remain unclear. Consistent with these evidences that MMP14 and Snail have been implicated in tumor development and progression, our study showed MMP14 and Snail were enriched in the primary GC tissues that inversely correlated to miR-22 levels. It is probable that the upregulation of MMP14 or Snail by suppression of miR-22 contributed to tumor progression in GC. MMP14 and Snail regulation by miR-22 was also examined in GC cell lines by western blotting and the luciferase reporter assay. Intriguingly, our mechanistic and functional data permit us to better appreciate the functional role of MMP14 and Snail in GC. Its expression positively regulated GC cells migration and invasion. Our results also indicated that MMP14 or Snail knockdown suppressed GC cells migration and invasion, which phenocopied the effects of miR-22 overexpression *in vitro*, and ectopic expression of MMP14 or Snail restored the effects of miR-22 on cell migration and invasion in GC cells. These data clearly demonstrated that MMP14 and Snail contribute to cell invasion and migration in GC and were direct and functional targets of miR-22.

**Figure 7:**
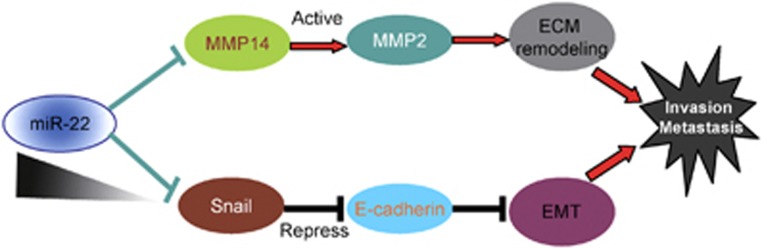
A schematic model depicting miR-22 downregulation promoted invasion and metastasis in GC cells. MiR-22, which was downregulated in GC, promotes GC invasion and metastasis by upregulating MMP14, causing MMP2 activation, and then inducing ECM remodeling. On the other hand, miR-22 downregulation promotes GC invasion and metastasis by upregulating Snail, causing E-cadherin downregulation and then inducing EMT

In conclusion, we identified that miR-22 is a potent tumor suppressor in GC. MiR-22 downregulation promotes GC invasion and metastasis by upregulating MMP14, causing MMP2 activation and then inducing ECM remodeling (Figure 7). On the other hand, miR-22 downregulation promotes GC invasion and metastasis by upregulating Snail, causing E-cadherin downregulation and then inducing EMT (Figure 7). To the best of our knowledge, this is the first study to demonstrate that the miR-22/MMP14/Snail axis regulates the proliferation, migration and invasion of GC cells. These findings provide a better understanding of the development and progression of GC and may be an important implication for future therapy of the GC.

## Materials and Methods

### Clinical samples

Sixty-one fresh GC tissue samples from GC patients, and their matched adjacent non-tumor gastric mucosal tissues (>5 cm laterally from the edge of tumor region) were obtained from the Southwest Hospital of Third Military Medical University (Chongqing, China). The samples had been clinically and histopathologically diagnosed according to the World Health Organization criteria between 2010 and 2012 (Table 1). Tumor and non-cancerous tissues were confirmed histologically by hematoxylin and eosin staining. All samples were collected from consenting individuals according to the protocols approved by the Ethics Review Board at Third Military Medical University.

### Cell culture

GC cell lines, including SGC-7901, HGC-27, AGS and BGC-823, were obtained from American Type Culture Collection (ATCC, Manassas, VA, USA). Cell culture was performed as described in previous studies.^15, 16^

### Microarray analysis

Microarray analysis was done as previously described.^15^ Briefly, the total RNA from above-mentioned GC tissues and non-tumor gastric mucosal tissues was labeled and hybridized following the manufacturer's protocol. Scanning was performed on GeneChip Scanner 3000 7G, signals were extracted and the subsequent data processing was performed using Affymetrix Transcriptom Analysis Console (Affymetrix Technologies, Santa Clara, CA, USA). The threshold we used to screen differentially expressed mRNAs with statistical significance is fold change ⩾1.5 and a *P*-value ⩽0.05.

### RNA isolation and qRT-PCR

Total RNA was extracted from the cultured GC cells and tissues harvested by Trizol reagent (Invitrogen, Carlsbad, CA, USA). To measure the level of miR-22, qRT-PCR was performed by using Taqman probes (Invitrogen) in the Bio-Rad CFX96 real-time PCR system (Bio-Rad, Hercules, CA, USA) according to the manufacturer's instruction. The data were normalized using the endogenous U6 snRNA. For MMP14 and Snail mRNA detection, reverse transcription was performed using the PrimeScript RT Master Mix (Perfect Real Time, TaKaRa, Dalian, China). Quantitative PCR was performed using SYBR Premix ExTaq II (TliRNaseH Plus) (TaKaRa) in Bio-Rad CFX96 real-time PCR system. Sense and antisense primers for MMP14 (178 bp) and Snail (234 bp) were 5′-TCGGCCCAAAGCAGCTTC-3′ and 5′-CTTCATGGTGTCTGCATCAGC-3′, and 5′-GGACTCTTGGTGCTTGTGGA-3′ and 5′-GGACTCTTGGTGCTTGTGGA-3′, respectively. Glyceraldehyde-3-phosphate dehydrogenase (GAPDH; sense primer: 5′-GAAGGTGAAGGTCGGAGTC-3′ and antisense primer: 5′-GAAGATGGTGATGGGATTTC -3′) gene was used as gene internal controls. Cycling conditions were 95 °C for 5 min, followed by 39 cycles of 95 °C for 15 s, 60 °C for 20 s and 72 °C for 10 s. Specificity of amplification products was confirmed by melting curve analysis. The 2^−ΔΔCT^ method was used in the analysis of PCR data.

### Cell proliferation assay

To measure the effect of miR-22 mimics, anti-miR-22 inhibitor on cellular proliferation rates, SGC-7901, and AGS cells were seeded at a density of 10^4^ per well in 96-well plates, respectively. The SGC-7901 cells were transfected with miR-NC, or miR-22 mimics, and AGS cells were transfected with miR-NC or anti-miR-22. Proliferation rates were analyzed using Cell Counting kit 8 (Dojindo Laboratories, Kumamoto, Japan) at 24, 48, 72-h posttransfection, and quantification was done on a microtiter plate reader (Bio-Rad) according to the manufacturer's protocol.

### Cell invasion and migration assays

SGC-7901 and HGC-27 cells were grown to 50–70% confluence and transfected with miR-NC, miR-22 mimics, si_con, si_MMP14#1, si_MMP14#2, si_Snail#1, si_Snail#2, pcDNA3.1 vector, pcDNA3.1-MMP14 vector or pcDNA3.1-Snail vector, co-transfected with miR-22 mimics and pcDNA3.1-MMP14, or co-transfected with miR-22 mimics and pcDNA3.1-Snail, respectively. AGS cells were transfected with miR-NC or anti-miR-22 inhibitor. Twenty-four hours posttransfection for invasion assay, cells were seeded onto a Matrigel-coated membrane matrix (BD Biosciences, San Jose, CA, USA) present in the insert of a 24-well culture plate (Costar, Corning, NY, USA). In the lower chamber, 500 *μ*l DMEM with 10% fetal bovine serum was added as chemoattractant. After 24 h, the noninvading cells were gently removed with a cotton swab. Invasive cells located on the lower surface of chamber were stained with the 0.1% crystal violet and counted under a microscope in five predetermined fields (× 200). The procedure for the cell migration assay was similar to the cell invasion assay, except that the transwell membranes were not precoated with matrigel. Cells adhering to the lower surface were counted the same way as the cell invasion assay. All assays were independently repeated at least three times.

### Constructs, reagents and assays

The 3′UTRs of the human MMP14 and Snail were cloned in between the *Spe*I and *Hind*III sites of pGL-3 (Promega, Madison, WI, USA). These vectors were named wild-type 3′UTR. Mutations of their 3′UTR sequence were created by using a Quick Change Site-Directed Mutagenesis kit (SBS Genetech, Beijing, China) and named as mutant 3′UTR. The mutant 3′UTR of MMP14 and Snail was served as a control. HEK293T cells were seeded onto 24-well plates (1 × 10^5^ cells per well) the day before transfections. Cells were transfected with the firefly luciferase reporter plasmid including the wild-type or mutant 3′UTR of MMP14 or Snail (50 ng per well), and pRL-TK Renilla luciferase reports (10 ng per well), and then the cells were transfected miR-22 mimics or miR-NC (50 nM). Cell lysates were prepared with Passive Lysis Buffer (Promega) 48 h after transfection, and luciferase activities were measured by using the Dual Luciferase Reporter Assay (Promega). The firefly luciferase activity was normalized to the renilla luciferase activity. For constructing pcDNA3.1-MMP14 and pcDNA3.1-Snail vectors, homo sapiens full open reading frame cDNA clones for MMP14 and Snail was transcribed, and the product was amplified by using primers with flanking *Spe*I and *Hind*III restriction enzyme digestion sites, followed by the DNAs were inserted into pcDNA3.1 vector (Invitrogen). All constructs were confirmed by DNA sequencing.

### Oligonucleotides and transfection

MiR-NC, MiR-22 mimics and anti-miR-22 were obtained from RIBOBIO (Guangzhou, China) and transfected with Lipofectamine 2000 (Invitrogen) in AGS, SGC-7901 or HGC-27 cells at a final concentration of 50 nM. siRNAs (specifically for MMP14 and Snail) and control siRNA were synthesized by RIBOBIO and transfected into SGC-7901 or HGC-27 cells (100 nM) using Lipofectamine 2000 (Invitrogen). Tansfection of empty vectors pcDNA3.1, pcDNA3.1-MMP14 and pcDNA3.1-Snail vectors was via Lipofectamine 2000 (Invitrogen). Agomir-NC and agomir-22 were synthesized by RIBOBIO. Transfection of oligonucleotides was performed using ribo*FECT* CP (RIBOBIO). In brief, agomir-NC and agomir-22 were added to culture media to a final concentration of 5 nmol/ml. Cells were grown in normal culture media to a 70% confluent state and were then treated with agomir-containing culture media for 48–72 h.

### *In vivo* tumor xenograft studies and metastasis assays

The animal studies were approved by the Institutional Animal Care and Use Committee of Third Military Medical University. Female athymic BABL/c nude mice (5–6 weeks old) were used for animal studies. Subcutaneous tumor growth assays were performed as previously described.^15^ In brief, 2 × 10^6^ SGC-7901 cells transfected with agomir-NC (5 *μ*M) or agomir-22 (5 *μ*M), respectively, were suspended in 200 *μ*l phosphate-buffered saline for each mouse and were injected subcutaneously into the axillary fossae of the female nude mice, eight mice per group. Tumor diameters were measured every 7 days. At 35 days after injection, mice were killed and tumors were weighted after necropsy. Tumor volume was calculated according to the standard formula (volume= length × width^2^ × 1/2). A peritoneal injection model was used for peritoneal dissemination assays, 1 × 10^6^ SGC-7901 cells transfected with agomir-NC or agomir-22, respectively, were injected into the abdominal cavity of nude mice (six per group). Four weeks after injection, the mice were killed and the macroscopic nodules in abdominal cavity of the mice were counted. A tail vein injection model was used for pulmonary metastasis assays as previously described,^15^ 1 × 10^6^ SGC-7901 cells transfected with agomir-NC (5 *μ*M) or agomir-22 (5 *μ*M), respectively. The cells were injected into the lateral tail veins of each anesthetized nude mice (10 per group). Five weeks after injection, the mice were killed. Lungs were fixed with phosphate-buffered neutral formalin before paraffin embedding, and 5-μm sections were stained with hematoxylin and eosin. The metastases were counted in a double-blind manner with the aid of a dissecting microscope (Nikon, Tokyo, Japan).

### Western blot assay

Western blot was carried out as described with rabbit polyclonal MMP14, Snail and E-cadherin antibodies (1 : 1000; Abcam, Cambridge, UK), as well as with mouse monoclonal MMP2 antibody (1 : 1000; Abcam). Mouse monoclonal GAPDH antibody (1 : 2000; Cell Signaling Technology, Shanghai, China) was used as internal reference. A horseradish peroxidase-conjugated anti-rabbit or anti-mouse immunoglobulin-G antibody was used as the secondary antibody (1 : 5000; Jackson Immuno-Research Laboratories Inc., West Grove, PA, USA). Signals were detected using enhanced chemiluminescence reagents (Pierce, Rockford, IL, USA).

### Statistical analysis

All data were analyzed using GraphPad Prism 5.0 (Graphpad Software Inc., La Jolla, CA, USA). Two-tailed Student's *t*-test was used to determine the differences between groups. The survival analysis using the Kaplan–Meier method was performed by the log-rank test. The relationships between miR-22 and MMP14, or Snail expression level were analyzed by correlation coefficients and linear regression analysis. Tumor lung metastases were analyzed by Fisher's exact test. *P*⩽0.05 was considered statistically significant.

## Figures and Tables

**Table 1:** Clinical and pathological characteristics of included patient samples

**Variable**	**Gastric cancer,** ***N*=61**
*Gender*	
Male	39 (64)
Female	22 (36)
	
*Age*	
Median (range)	57 (32–76)
⩾55	32 (53)
<55	29 (47)
	
*Tumor location*	
Body	30 (49)
Antrum	21 (35)
Cardia	8 (13)
Other	2 (3)
	
*Histology*	
Adenocarcinoma	48 (79)
Mucinous adenocarcinoma	12 (20)
Signet ring cell cancer	1 (1)
	
*TNM stage*	
I	10 (17)
II	13 (21)
III	27 (44)
IV	11 (18)
	
*Lymph node status*	
Metastasis	46 (75)
No metastasis	15 (25)

## Abbreviations

GC: gastric cancer
ECM: extracellular matrix
MMPs: matrix metalloproteinases
EMT: epithelial-to-mesenchymal transition
qRT-PCR: quantitative real-time PCR
TET: Ten eleven translocation
TOB1transducer of ERBB2: 1
3′UTR: 3′ untranslated regions
siRNA: small interfering RNA
GAPDH: glyceraldehyde-3-phosphate dehydrogenase
