# Supplementary material for: MicroRNA-22 inhibits tumor growth and metastasis in gastric cancer by directly targeting MMP14 and Snail
Source: Cell Death Dis. 2015 Nov 26;6(11):e2000–. doi: 10.1038/cddis.2015.297 (PMC4670920; doi:10.1038/cddis.2015.297)
Supplement: Supplementary Information [file cddis2015297x1.doc]

Supplementary information for the manuscript:

**MicroRNA-22 inhibits tumor growth and metastasis in gastric cancer by directly targeting MMP14 and Snail**

**Q-F Zuo^1,3^, L-Y Cao^1,3^, T Yu^1^, L Gong^1^, L-N Wang^1^, Y-L Zhao^2^, B Xiao^*, 1^ and Q-M Zou^*, 1^**

^1^National Engineering Research Center of Immunological Products, Department of Microbiology and Biochemical Pharmacy, College of Pharmacy, Third Military Medical University, Chongqing 400038, PR China. ^2^General Surgery and Center of Minimally Invasive Gastrointestinal Surgery, Southwest Hospital, Third Military Medical University, Chongqing 400038, PR China.

^*^Correspondence: Associate Professor B Xiao or Professor Q-M Zou

Department of Microbiology and Biochemical Pharmacy, College of Pharmacy, Third Military Medical University, NO.30, Gao Tan Yan Road, Sha Ping Ba District, Chongqing 400038, China.

E-mail: Binxiaotmmu@163.com or [Qmzou2007@163.com](mailto:Qmzou2007@163.com)

^3^These authors contributed equally to this work.

**Supplementary Figure Legends**

**Supplementary Figure 1. Overexpression of miR-22 inhibits GC growth in tumor xenograft studies.** Photographs of mice injected with agomir-NC-SGC-7901 or agomir-22-SGC-7901. 2× 10^6^ SGC-7901 cells transfected with agomir-NC (5μM) or agomir-22 (5μM), respectively, were suspended in 200μL phosphate-buffered saline for each mouse and were injected subcutaneously into the axillary fossae of the female nude mice. At 35 days after injection, mice were killed and photographed (Nikon, Tokyo, Japan).
