# Supplementary figures and images for: MicroRNA-22 inhibits tumor growth and metastasis in gastric cancer by directly targeting MMP14 and Snail
Source: Cell Death Dis. 2015 Nov 26;6(11):e2000–. doi: 10.1038/cddis.2015.297 (PMC4670920; doi:10.1038/cddis.2015.297)

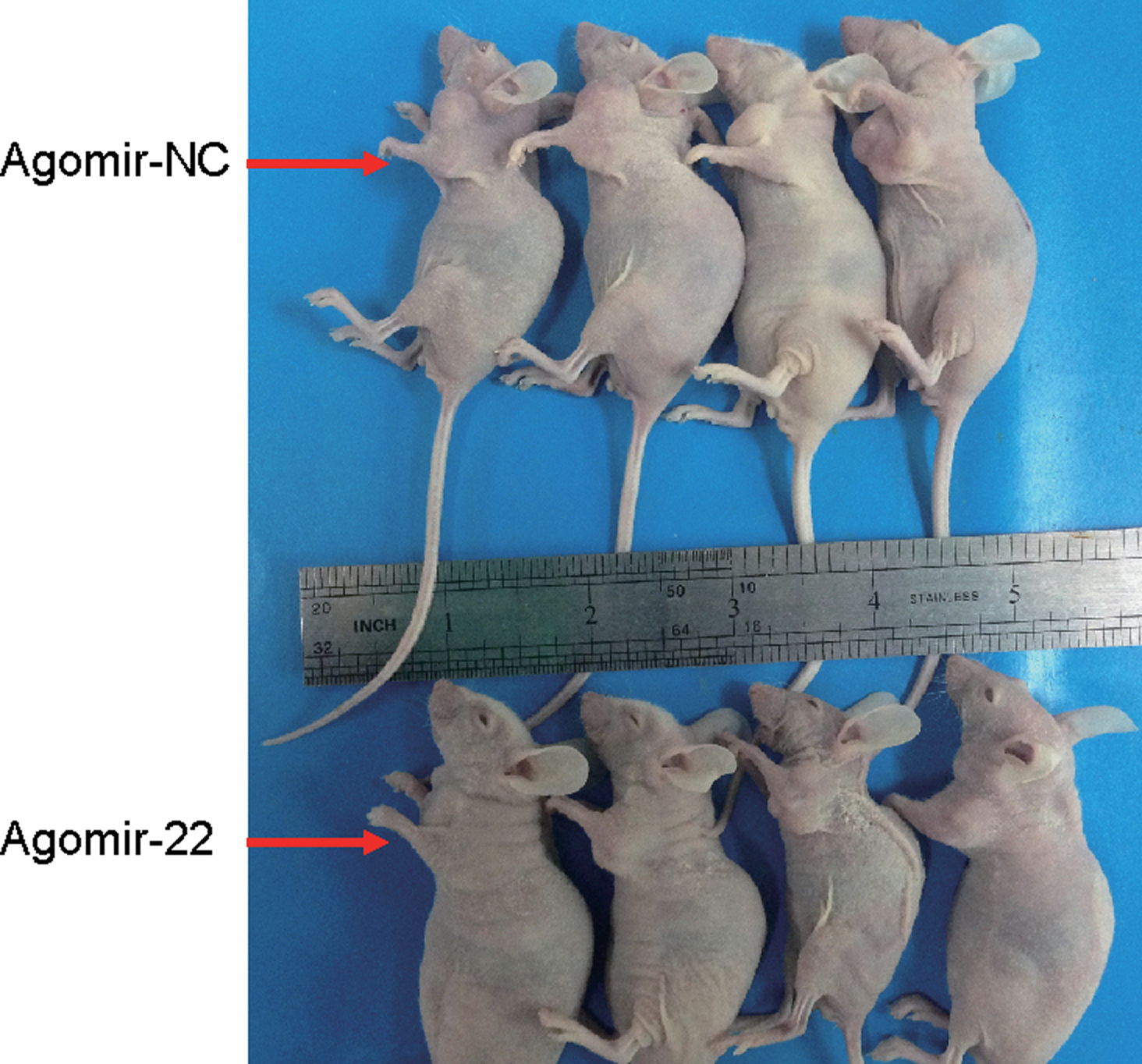

Supplement: Supplementary Figure [file cddis2015297x2.tif]
